# Clinical Outcomes from the Alaska Native Tribal Health Consortium Colorectal Cancer Control Program: 2009–2015

**DOI:** 10.3390/ijerph21050552

**Published:** 2024-04-26

**Authors:** Sarah H. Nash, Elizabeth Verhage, Christie Flanagan, Donald Haverkamp, Elena Roik, Garrett Zimpelman, Diana Redwood

**Affiliations:** 1 Department of Epidemiology, College of Public Health, University of Iowa, Iowa City, IA 52242, USA; 2 Holden Comprehensive Cancer Center, University of Iowa, Iowa City, IA 52242, USA; 3 State Health Registry of Iowa, College of Public Health, Iowa City, IA 52242, USA; 4 Alaska Native Epidemiology Center, Alaska Native Tribal Health Consortium, Anchorage, AK 99508, USA; 5 Centers for Disease Control and Prevention, Division of Cancer Prevention and Control, Albuquerque, NM 87110, USA

**Keywords:** Alaska Native, cancer screening, colonoscopy, quality improvement, quality metrics, screening quality, screening outcomes

## Abstract

The Alaska Native Tribal Health Consortium (ANTHC) participated in the United States Centers for Disease Control and Prevention Colorectal Cancer Control Program (CRCCP) from 2009 to 2015. We conducted a descriptive evaluation of ANTHC CRCCP demographics, quality measures, and clinical outcomes, including screening methods employed within the program and screening outcomes. There were 6981 program screenings completed, with the majority (81.3%) of people screened in the 50–75 year age group. Colonoscopy was the primary screening test used, accounting for 6704 (96.9%) of the screening tests. Quality of colonoscopy was high: adequate bowel preparation was reported in 98.2% of colonoscopies, cecal intubation rate was 98.9%, and the adenoma detection rate was 38.9%. A high proportion (58.9%) of colonoscopies had an initial finding of polyps or lesions suspicious for cancer; 41.2% of all colonoscopies had histological confirmation of either adenomatous polyps (40.6%) or cancer (0.5%). The ANTHC CRCCP successfully increased CRC screening among American Indian and Alaska Native peoples living in Alaska; this was achieved primarily through high-quality colonoscopy metrics. These data support a continued focus by the Alaska Native Tribal Health Consortium and its tribal health partners on increasing CRC screening and reducing cancer mortality among Alaska Native peoples.

## 1. Introduction

Alaska Native Tribal health organizations, collectively called the Alaska Tribal Health System, have recognized colorectal cancer (CRC) as a major health priority and as a result have focused substantial attention on increasing awareness of, access to, and use of CRC screening [1,2,3]. This focus is driven by the disproportionately high incidence of CRC among Alaska Native peoples [4], who also have a high prevalence of precancerous colorectal polyps [5,6]. CRC is particularly suitable for population-based screening because most cases develop from pre-malignant polyps which slowly develop into CRC over a period of 10–15 years [7]. CRC screening tests currently recommended by the United States Preventive Services Task Force for average-risk individuals aged 45–49 years (B grade) and 50 years and over (A grade) include annual high-sensitivity guaiac-based fecal occult blood test (gFOBT) or fecal immunochemical test (FIT), stool DNA-FIT every 1–3 years, computed tomography colonography or flexible sigmoidoscopy every 5 years, or colonoscopy every 10 years [8]. Through screening methods such as colonoscopy, polyps can be detected and removed before they become malignant, leading to reduced CRC incidence and mortality [9]. Where cancer is detected by screening, it is often detected earlier [10], when treatments are more likely to be curative, less invasive, and have less severe effects on quality of life. Indeed, 5-year survival for early-stage CRC diagnosis is around 90%, compared to approximately 14% for late-stage diagnosis [7].

Multi-level CRC screening efforts within the Alaska Tribal Health System have included education and outreach programs [3]; patient navigation and reminder systems [1]; development of a first-degree relative family outreach program [11]; and systems and policy changes. For example, in 2013 the Alaska Native Medical Center began screening Alaska Native peoples for CRC starting at age 40 years [12]. In 2009–2015, the Alaska Native Tribal Health Consortium (ANTHC) received funding from the Centers for Disease Control and Prevention’s (CDC) Colorectal Cancer Control Program (CRCCP), the goal of which was to increase high-quality population-based CRC screening among average-risk, uninsured and underinsured persons 50 years of age and older (screening of all adults ages 50–75 was the national recommendation at that time) [13,14,15,16,17]. The ANTHC CRCCP was one of three tribal health organizations funded in Alaska under the national CRCCP. These combined efforts to increase CRC screening within the Alaska Tribal Health System successfully increased screening prevalence among Alaska Native peoples. Screening prevalence increased from less than 40% in 1999 to 47% in 2008, before CRCCP efforts began, to 68% in 2016, after the 2009–2015 CRCCP cycle [18].

Even with the substantial increase in screening prevalence among Alaska Native peoples as a result of these efforts, CRC mortality is increasing among this population despite marginal decreases in incidence among those aged 50–75 years [19,20,21]. Colonoscopy and stool-based test quality is closely linked with screening effectiveness for reducing mortality [22,23,24,25]. Further, timely follow-up after initial screening tests is critical to early detection of and reduced mortality from CRC. Thus, understanding the quality and outcomes of CRC screening among Alaska Native peoples may provide insight into how to improve CRC outcomes in this population. Here, we evaluated CRC screening and quality metrics from CRC screenings completed through the ANTHC CRCCP from 2009 to 2015 [15].

## 2. Methods

### 2.1. Ethical and Tribal Approval

This program evaluation was approved by the Alaska Area Institutional Review Board (IRB #2019-01-004) and the University of Iowa Institutional Review Board (IRB #202203676). Tribal approval for this study and publication of study results was received from ANTHC and Southcentral Foundation.

### 2.2. Study Setting

**Figure 1:**
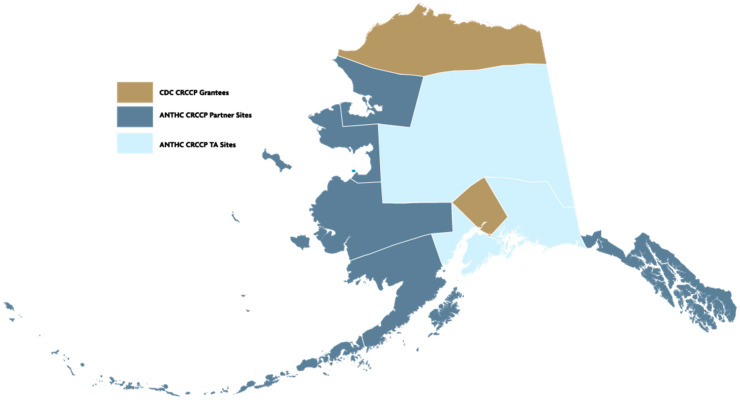
Map of Alaska indicating Tribal Health Regions served by CDC CRCCP direct grantees, by ANTHC CRCCP partner sites, and ANTHC CRCCP technical assistance sites, 2009–2015. ANTHC: Alaska Native Tribal Health Consortium; CRCCP: Colorectal Cancer Control Program.

This study reports data from the ANTHC CRCCP for Alaska Native and American Indian people living in Alaska. The ANTHC CRCCP had formal partnerships with eight regional tribal health organizations to increase screening statewide. The ANTHC CRCCP also provided technical assistance to an additional three regional tribal health organizations (Figure 1). Information on the national CRCCP has been previously reported [16].

### 2.3. Data Elements

For each person served by the CRCCP, grantees collected a standardized set of CRC clinical data from the medical record outlined in the CDC CRCCP Data User’s Manual [26,27]. Demographic information included age, sex, personal history of colorectal polyps or CRC, prior CRC screening before CRCCP enrollment, income, family history of CRC, and CRCCP eligibility, i.e., those whose annual household income was ≤250% of the federal poverty level and were uninsured or underinsured for CRC screening services. “Underinsured” was defined by the program as not having insurance coverage for preventive services or having only Indian Health Service health care benefits.

Our evaluation of screening outcomes and quality focused on several metrics. First, we report type of screening received (colonoscopy, FIT, or other, which may have included double contrast barium enema [DCBE] or flexible sigmoidoscopy). FOBT is not typically used among the Alaska Native population, because of the high number of false-positive results associated with a high prevalence of Helicobacter pylori-associated hemorrhagic gastritis [28]. For all screenings, we report screening outcomes (e.g., incomplete, not returned, negative/normal, polyp/lesion, or other finding not suspicious for cancer), stratified by screening type. Among those who completed FIT screening, we report (1) the outcomes of FIT testing and follow-up recommendations, and (2) where follow-up was recommended, whether a colonoscopy was received. For those who had a biopsy or polypectomy performed during colonoscopy, we report the most severe histology, including the number and size of polyps. For those who received a colonoscopy and for whom bowel preparation was considered by the endoscopist to be adequate for a complete exam (yes/no) [26], we calculated cecal intubation rate and scope withdrawal time. Adenoma detection rate (ADR) was calculated as the percentage of colonoscopies in which at least one adenoma or cancer was found among people who were aged 40 years and above, and who had no personal or family history of CRC, no CRC symptoms, adequate bowel preparation, and complete cecum intubation [15].

Within the ANTHC CRCCP, an individual could have more than one screening method or procedure per screening cycle; cycles were defined as an initial CRC screening test, continuing through any additional tests or procedures required for evaluation following an abnormal or incomplete test. Cycles ended when a final diagnosis or outcome was determined. Thus, at the end of each cycle, a person received one final screening result. Within the program period, 2009–2015, an individual could have more than one screening cycle; demographic analyses are presented at the individual level, whereas quality metrics are reported at the individual-cycle level. Given incomplete reporting for people who did not receive or refused screening, we focused our evaluation only on those who received screening.

### 2.4. Statistical Analysis

Descriptive statistics (frequencies, proportions) were calculated. All analyses were conducted using SPSS version 28.0.1.0 (IBM Corp. Released 2021. IBM SPSS Statistics for Mac, Version 28.0.1.0, Armonk, NY, USA: IBM Corp).

## 3. Results

The majority of individuals screened by the program (81.3%) were aged 50–75 years, with 17.0% younger than 50 years and 1.7% older than 75 years. Most individuals screened were female (56.2%), had an annual household income ≤250% of federal poverty level (70.7%), and were un- or under-insured (79.7%). Half (50.1%) had a prior screening completed, and half had never been screened before. Further, 29.6% had a family history of CRC, and 29.9% a personal history of adenomatous polyps (Table 1).

There were 6981 screenings performed as part of the ANTHC CRCCP. Of these, 6765 were initial screenings (i.e., not follow-ups to an initial abnormal test). Of the initial screenings only, 96.9% were colonoscopy, 2.8% FIT, and 0.3% other tests (flexible sigmoidoscopy or DCBE). Table 2 gives screening outcomes for the 6765 initial screenings conducted as part of the ANTHC CRCCP, stratified by screening type. Among the 188 people who completed a FIT test as their initial screening, 30 (16.0%) had an abnormal result. Of those with abnormal FIT results, 28 (93%) received a colonoscopy to complete diagnosis. Of the 6557 colonoscopies performed as initial screening tests, 3863 (58.9%) had a finding of polyps or lesions suspicious for cancer; of those, 3692 were recommended no additional follow-up, 91 were recommended a follow-up colonoscopy to complete the screening cycle, 49 were recommended surgery, 13 DCBE, 16 sigmoidoscopy, and <5 another approach.

Table 3 shows quality measures for all colonoscopies (*n* = 6704) conducted as part of the ANTHC CRCCP (either as initial screenings or follow-up tests). The majority of colonoscopies (74.8%) were for screening, and a biopsy was performed in 60.0% of exams. Over 98% of the colonoscopies had adequate bowel preparation, and among that subgroup, almost 99% were complete to the cecum. There were few complications (0.5% procedures), and overall ADR was 38.9%. Among those with adequate bowel preparation and measured withdrawal time, the mean scope withdrawal time was 14.6 min (standard deviation [SD] ± 12.0).

Results from colonoscopies conducted as part of the ANTHC CRCCP are reported in Table 4. Of the 6704 colonoscopies performed as part of the program (either as initial screenings or follow-up tests), 46.0% had normal histology/no findings; 10.7% had hyperplastic polyps; and 41.3% had a finding of adenomatous polyp/CRC. Of the final diagnoses from all screening cycles (*n* = 6770), 0.5% of screening cycles (*n* = 36) resulted in a diagnosis of cancer, 39.9% (*n* = 2701) resulted in a finding of adenomatous polyp with or without high-grade dysplasia, while less than half (48.1%; *n* = 3258) were normal.

## 4. Discussion

CRC screening is a priority for the ANTHC and its tribal health partners, in part because of the high prevalence of CRC and polyps observed among Alaska Native peoples [4,5]. Despite increases in screening prevalence over time [29], CRC mortality is increasing in this population [20]. Therefore, it is important to understand whether colonoscopy quality could in part explain this disconnect. In this evaluation of CRC screening quality and outcomes from the 2009–2015 ANTHC CRCCP, we observed the following: that colonoscopy was performed more often than other screening methods (e.g., FIT); high adherence to quality metrics for colonoscopy, including adequate bowel preparation and cecal intubation rate; and high prevalence of screening-detected polyps and cancers. These findings indicate high screening quality for colonoscopies delivered as part of the ANTHC CRCCP. Our evaluation also revealed a high percentage of individuals with a family history of CRC or a personal history of adenomatous polyps and an overall increased risk of CRC compared to other populations. Of note, a large proportion of people served by the ANTHC CRCCP were outside the nationally recommended guideline age (i.e., 50–75 years), because of a high proportion meeting high-risk status (i.e., previous polyp history, family history). Further, over 53% of screenings completed were abnormal, requiring more frequent surveillance follow-up for these individuals. This has important implications for health service planning and care delivery, including ensuring availability of colonoscopies for Alaska Native peoples age-eligible for screening and surveillance.

Almost all screenings completed by the ANTHC CRCCP during 2009–2015 were colonoscopies (96.9%). This is in contrast to CRC screenings provided by the other 29 national CRCCP sites funded by the CDC during this program cycle, of which 39% were colonoscopies, 21% FOBT, and 40% FIT [17]. This discrepancy might be driven by differences in demographics and risk profiles between the ANTHC and other CRCCP sites. For example, there may have been differences in the proportion of participants with a family history of cancer, and those with a personal history of cancer or polyps, and/or physician or patient preference. Yet, our finding that most screenings in the ANTHC CRCCP were colonoscopies is in line with the Alaska Native Medical Center’s CRC Screening Guidelines used throughout the Alaska Tribal Health System, which recommends colonoscopy as the preferred screening test, with stool tests available for patients who decline colonoscopy [12]. These guidelines were enacted in direct response to the high incidence of and mortality from CRC among Alaska Native peoples [30], and the high prevalence of polyps/CRC, as evidenced by this study and others [5]. Indeed, in our study, 41.2% had a finding of adenomatous polyp/CRC, which is comparable to previous studies in this population, in which 34.4% of individuals aged 40–49 years and 67.1% of individuals aged ≥50 years were found to have adenomatous polyps [6]. While FIT was added to the Alaska Native Medical Center’s CRC Screening Guidelines starting in 2013, the guidelines encourage preferential use of colonoscopy where available and where individuals are willing to undergo the procedure; our data suggest that colonoscopy remained the primary screening test during the ANTHC CRCCP (2009–2015).

High-quality colonoscopy is central to screening effectiveness and, in turn, the prevention of CRC [22,23]. The American Society for Gastrointestinal Endoscopy/American College of Gastroenterology Task Force on Quality in Endoscopy recommends the following intra-procedure endoscopic quality indicators to ensure high-quality examinations: bowel preparation adequacy (≥85%), screening cecal intubation rate with photo documentation (≥95%), and scope withdrawal time (≥6 min average) [31]. Finally, recommended minimum thresholds for adenoma detection in asymptomatic, average-risk screening individuals (screening) are ≥25% overall (≥30% in men and ≥20% in women) [22]. The ANTHC CRCCP met all quality indicators, including 98% bowel preparation adequacy, 99% cecal intubation (98.9% for screening colonoscopies, and 99.3% for surveillance colonoscopies), and an average scope withdrawal time of 14.6 (SD 12.0) minutes. Our overall ADR of 38.9% in the ANTHC CRCCP was substantially higher than national performance targets. Together, these findings demonstrate that Alaska Native peoples are receiving high-quality colonoscopies within the Alaska Tribal Health System, which is important to support prevention in this population at increased risk.

Recent national benchmarking data indicate that the ADR has increased over time in the U.S. and was recently 39% among a large national U.S. sample standardized to the 2000 U.S. population [32]. Findings from all CRCCP grantees during this time period indicated an ADR of 36.0% among average-risk men, and 25.7% among average-risk women. This is substantially lower than the ADR presented herein for Alaska Native program participants (45.3% among men, 33.9% among women). However, our findings were slightly lower than reported in a recent study examining the ADR among Alaska Native peoples living in Interior Alaska (45.0% overall: 43.0% among women and 47.1% among men) [6]. Reasons for differences in ADR between these studies/programs may include differences in demographic or risk profile, such as the number of men/women screened (ADR is typically higher among men), or the proportion of those screened with a family or personal history of CRC/polyps (ADR is expected to be higher among those with greater-than-average risk).

This study has several strengths and limitations that should be considered in the interpretation of its findings. The primary strength of this study is that the ANTHC CRCCP is the largest database of screening information available for Alaska Native peoples; therefore, these data represent the most comprehensive study of CRC screening outcomes for this increased-risk population. Further, because the ANTHC CRCCP followed national CRCCP data reporting requirements, our results are directly comparable to previously reported results for that program [15]. Study limitations include that grant program funding and thus data collection ended in 2015; thus, screening outcomes among Alaska Native peoples may have changed since the program’s end. Although colonoscopy quality measures were high, we were not able to delve into key considerations that may influence measures. For example, the ADR is strongly mediated by the experience of the endoscopist [31,33]; however, we did not have information on the number of colonoscopies performed per endoscopist to assess associations in this population. We were not able to analyze data by region (tribal health organization) due to small cell sizes for some analyses, which may be important given known differences in CRC mortality across Alaska [30]. Finally, this program evaluation was not able to evaluate prevalence of known risk and protective factors for CRC among this Alaska Native population, as these data were not collected by the CRCCP. Collection of additional data, such as body mass index, dietary patterns, presence of comorbid conditions, and water quality, could help in understanding risk and outcomes of CRC among Alaska Native peoples, and may be the focus of future research.

## 5. Conclusions

In this study, we present the results of an evaluation of CRC screening methods and outcomes in the ANTHC CRCCP, demonstrating high use of colonoscopy for screening, high quality of colonoscopy procedures, and high prevalence of polyps/CRC among Alaska Native peoples. We conclude that colonoscopy quality is unlikely to explain why CRC mortality is increasing among Alaska Native peoples, despite increases in CRC screening. These data support a continued focus by the ANTHC and its Alaska Tribal Health System partners on CRC screening for cancer prevention and mortality reduction.

## Figures and Tables

**Table 1:** Demographic characteristics of Alaska Native individuals in the Alaska Native Tribal Health Consortium Colorectal Cancer Control Program, 2009–2015, *n* = 6436.

Demographics	*n* (%)
Age (years)	
<50	1092 (17.0)
50–75	5230 (81.3)
76+	109 (1.7)
Missing	5 (0)
Sex	
Female	3619 (56.2)
Male	2817 (43.8)
Program eligible income ^a^	
Yes	4549 (70.7)
No	1837 (28.5)
Unknown/missing	50 (0.8)
Program eligible insurance ^b^	
Yes	5127 (79.7)
No	1293 (20.1)
Unknown/missing	16 (0.2)
Previous CRC screening	
Yes	3226 (50.1)
No	3201 (49.7)
Unknown	9 (0.1)
Personal history of adenomatous polyps	
Yes	1922 (29.9)
No	4448 (69.1)
Unknown	65 (1.0)
Family history of CRC	
Yes	1908 (29.6)
No	4354 (67.7)
Unknown	174 (2.7)

^a^ Annual household income ≤ 250% of the federal poverty level. ^b^ Defined as not having insurance coverage for preventive services or having only Indian Health Service health care benefits.

**Table 2:** Initial test outcomes and recommended follow-up within the Alaska Native Tribal Health Consortium Colorectal Cancer Control Program, 2009–2015, stratified by test modality, among first screening tests only, *n* = 6765.

Test	*n* (% Total)	Initial Results	Frequency (%)	Follow-Up Needed to Complete Diagnosis ^a^					
				None	Colonoscopy	Surgery	DCBE ^b^	Sigmoidoscopy	Other
FIT	188 (2.8)	Normal/negative	158 (84.0)	158	0	0	0	0	0
		Abnormal/positive	30 (16.0)	<5 ^c^	28	0	0	0	<5 ^c^
Colonoscopy	6557 (96.9)	Polyp(s) or lesion(s) suspicious for cancer	3863 (58.9)	3692	91	49	13	16	<5 ^c^
		Normal/negative	2469 (37.7)	2469	0	0	0	0	0
		Other finding not suggestive of cancer/polyps	121 (1.8)	114	<5^c^	<5^c^	<5^c^	0	0
		Inadequate/incomplete tests with no findings ^d^	104 (1.6)	20	42	<5^c^	34	<5^c^	5

^a^ Flexible sigmoidoscopies or DCBE (*n* = 20) not included in table due to small cell sizes. ^b^ DCBE: Double contrast barium enema. ^c^ Cell sizes < 5 suppressed to protect privacy. ^d^ Colonoscopies with inadequate preparation and/or cecum not reached.

**Table 3:** Colonoscopy quality measures for all colonoscopies performed by the Alaska Native Tribal Health Consortium Colorectal Cancer Control Program, 2009–2015, *n* = 6704.

Quality Metric	*n* (%)
Colonoscopy indication	
Screening	5017 (74.8)
Surveillance	1545 (23.1)
Unknown	142 (2.1)
Biopsy performed	
Yes	4024 (60.0)
No	2680 (40.0)
Adequate bowel preparation	
Yes	6589 (98.2)
No	111 (1.7)
Unknown	<5 ^c^ (0.1)
Cecal intubation, ^a^ screening colonoscopies (*n* = 5017)	4915 (98.0)
Cecal intubation, ^a^ surveillance colonoscopies (*n* = 1545)	1522 (98.5)
Complications	
Yes	33 (0.5)
No	6671 (99.5)
Adenoma detection ^b^	
Overall (*n* = 2773)	
No	1694 (61.1)
Yes	1079 (38.9)
Men (*n* = 1219)	
No	667 (54.7)
Yes	552 (45.3)
Women (*n* = 1554)	
No	1027 (66.1)
Yes	527 (33.9)
Scope withdrawal time, mean minutes (SD) (*n* = 5521)	14.6 (12.0)

^a^ Cecal intubation rate calculated for those with adequate bowel preparation. ^b^ Adenoma detection for screening colonoscopies with non-missing scope withdrawal time and adequate bowel preparation, where the cecum was reached, among individuals with no family or personal history of CRC and no CRC symptoms. ^c^ Cell sizes < 5 suppressed to protect privacy.

**Table 4:** Results from all colonoscopies (*n* = 6704) conducted as part of the Alaska Native Tribal Health Consortium Colorectal Cancer Control Program, 2009–2015.

Histology of Most Severe Polyp ^a,b^	*n* (%)
Adenocarcinoma, invasive	26 (0.4)
Adenoma with high-grade dysplasia (includes in situ carcinoma)	50 (0.7)
Adenoma with tubulovillous or villous histology	281 (4.2)
Adenoma, not otherwise specified	86 (1.3)
Adenoma, serrated	310 (4.6)
Adenoma, tubular	
1–2 tubular adenomas <10 mm	1269 (18.3)
3–4 tubular adenomas <10 mm	250 (3.7)
5–10 tubular adenomas <10 mm	91 (1.4)
All other tubular adenomas	444 (6.6)
Cancer, other	<5 ^c^ (0.1)
Hyperplastic polyps	716 (10.7)
Non-adenomatous polyp	79 (1.2)
Normal or other non-polyp histology	3081 (46.0)
Unknown/other lesions ablated, not retrieved or confirmed	17 (0.3)

^a^ Individuals without a biopsy were considered to have “normal” histology. ^b^ No size or number available for non-adenomatous polyps. ^c^ Cell sizes < 5 suppressed to protect privacy.

## Data Availability

Data are not available due to tribal research data restrictions. Researchers interested in these data may contact the corresponding author for more information on the process for requesting access to these tribal data.
